# Onco-STS: a web-based laboratory information management system for sample and analysis tracking in oncogenomic experiments

**DOI:** 10.1186/s13029-014-0025-z

**Published:** 2014-12-05

**Authors:** Mike Gavrielides, Simon J Furney, Tim Yates, Crispin J Miller, Richard Marais

**Affiliations:** Molecular Oncology Group, University of Manchester, Wilmslow Road, Manchester, M20 4BX UK; Applied Computational Biology and Bioinformatics Group, Cancer Research UK Manchester Institute, University of Manchester, Wilmslow Road, Manchester, M20 4BX UK

**Keywords:** Sample tracking, LIMS, Oncogenomic data, Cancer, Grails, Web application, Database

## Abstract

**Background:**

Whole genomes, whole exomes and transcriptomes of tumour samples are sequenced routinely to identify the drivers of cancer. The systematic sequencing and analysis of tumour samples, as well other oncogenomic experiments, necessitates the tracking of relevant sample information throughout the investigative process. These meta-data of the sequencing and analysis procedures include information about the samples and projects as well as the sequencing centres, platforms, data locations, results locations, alignments, analysis specifications and further information relevant to the experiments.

**Results:**

The current work presents a sample tracking system for oncogenomic studies (Onco-STS) to store these data and make them easily accessible to the researchers who work with the samples. The system is a web application, which includes a database and a front-end web page that allows the remote access, submission and updating of the sample data in the database. The web application development programming framework Grails was used for the development and implementation of the system.

**Conclusions:**

The resulting Onco-STS solution is efficient, secure and easy to use and is intended to replace the manual data handling of text records. Onco-STS allows simultaneous remote access to the system making collaboration among researchers more effective. The system stores both information on the samples in oncogenomic studies and details of the analyses conducted on the resulting data. Onco-STS is based on open-source software, is easy to develop and can be modified according to a research group’s needs. Hence it is suitable for laboratories that do not require a commercial system.

**Electronic supplementary material:**

The online version of this article (doi:10.1186/s13029-014-0025-z) contains supplementary material, which is available to authorized users.

## Background

The field of cancer genomics has been revolutionized by the development of high throughput sequencing technologies to survey tumour genomes [1]. Targeted and genome-scale sequencing and analysis have been conducted on DNA and RNA from multiple samples to detect aberrations in many tumour types [2-6]. Whole genomes and exomes are investigated to detect some or all of single nucleotide variations (SNVs), small insertions and deletions (indels), copy number alteration and structural variation. Messenger RNA and micro RNA expression are also analysed to obtain insights in gene expression differences in tumour cells [3-6].

Recently, large scale cancer genomics projects such as the International Cancer Genome Consortium (ICGC) [7] and the Cancer Genome Atlas (TCGA) [8] have been launched and are already beginning to bear fruit. In addition, the plummeting cost of next-generation sequencing has allowed smaller sequencing efforts, such as our own and others, to address questions in cancer genomics beyond the remit of the larger scale projects [9-13]. However, even smaller oncogenomic projects can produce data from different techniques and sequencing platforms that then undergo a variety of alignment and analysis procedures. Furthermore, more established oncogenomic experiments such as mRNA and DNA microarrays and array comparative genomic hybridization (aCGH) are often used in tandem with next generation technologies. Hence a sample from a patient may be surveyed by a number of different technologies within the same laboratory or study. Molecular biology laboratories need to store relevant information from the sequencing procedures and analysis to be able to track an experiment’s progress and specifications of each sample under study. The data management and sample tracking systems implemented by larger initiatives are often not available to smaller institutes or laboratories and information is sometimes stored in manually managed text or spreadsheet files that render access, search and management of the data tedious.

A number of Laboratory Information Management Systems (LIMS) for next generation sequencing experiments are freely available, including GNomEx [14], openBIS [15], Galaxy LIMS [16] and SeqBench [17]. However, none of these systems is designed specifically for oncogenomic experiments in which both array based and different sequencing technologies are used to analyse samples from tumour, germline, *in vitro* and xenograft models.

This work presents an open-source and platform independent web-based system that facilitates remote storage and retrieval of next-generation sequencing/oncogenomic experiment related information. The Sample Tracking System (Onco-STS) enables users to track DNA and RNA samples that have been sequenced or analysed in genomic studies. Onco-STS, which runs on a server, is accessible remotely by users and includes a database and a web interface through which users interact with the database.

## Methods

### Implementation

The system has been developed using the web development framework, Grails (http://www.grails.org/). Grails has a persistence engine built on Hibernate (http://www.hibernate.org/), and a straightforward Object Relational Mapping (ORM) that makes it ideal for the rapid development of database-backed web applications, since most database actions can be handled automatically without any explicit SQL or XML coding on the part of the programmer. The entire Grails project is wrapped in a *war* file with a single command and deployed in an Apache Tomcat servlet container. The application is easy to install and takes about 30 minutes. All the necessary files and instructions to run the application are included in the supplementary material submitted with this work. The system is protected by an authentication system and users must have an account to login to the service in order to use it. Users may also be granted different authorities according to the different privileges they may have. As the system is designed for individual laboratories and facilities within a research institute, registered users have access to the complete database, which means that any registered user can see all samples in the database and edit them if they have been assigned editing privileges. There are three different user account types: a “read-only” account for users that need only to access the data but will not be allowed to add new or alter the existing data, a “write” account for users that will be able to submit new samples and add new data for the existing ones and “administrator” account for the administrators of the system that in addition to updating privileges they will also be able to manage the users and their accounts.

Onco-STS also has an optional automatic mailing system that notifies the users and investigators about sample and experimental updates. If activated, only users and investigators associated with the updated sample will be informed. Mailing settings and user management are available in the Administration page. The homepage of Onco-STS is shown in Additional file 1: Figure S1.

## Results

### Sample tracking data

Data to be submitted to the database include the tumour or germline sample tissue information, nucleic acid extraction information, the different stages of the oncogenomic experiments that have been conducted on the samples, the projects that the samples are involved in and the sources from which the samples have been extracted.

Each sample belongs to a particular project that has a unique code and name and involves a number of investigators. A project can have multiple samples from the same or different sources (Additional file 1: Figure S2). Sample information includes the sample name, the type of sample (whether it is germline, a primary tumour, a metastasis, xenograft, cell line or allograft), the site and histology, surgery information and mouse generation and number in case of a xenograft/allograft. The metadata of the samples include the dates the sample was submitted to the database, the last time they were altered, the user who created the sample and all the users who have edited the sample or any other information related to the sample experiments. Storage information of the samples under various conditions is also supported (Additional file 1: Figures S3 & S4). To access samples of interest users can list all the samples of a project by navigating to the project page or they can use the search form to search for samples by their main attributes (e.g. name, type, site, histology, user that submitted the sample). A quick sample search at the home page is also available.

Information for six oncogenomic high-throughput experiments for each sample are stored in the system: whole genome sequencing (WGS), whole exome sequencing (WES), RNA sequencing, SNP array, mRNA microarray and aCGH (Additional file 1: Figure S4). Any data files such as FASTQ or BAM files should be saved separately outside Onco-STS. Onco-STS is designed only for sample and analysis metadata and not for hosting the actual sequencing and analysis data. Nevertheless, location and paths to these data can be saved in the sample experiment data under “Sequencing Data” section and “Results Location/Directory” in analysis section, thus multiple files for the same sample can be tracked.

i. *Whole Genome Sequencing;*
  The whole genome sequencing data include the genome library, the sequencing process and data - dates, sequencing centre, platform, number of lanes, insert and fragment size, location of results etc. – alignment, analysis and somatic analysis, which includes used algorithms, duplicates, mapping information, results locations, dates performed etc. For the sequencing part quality control information for each lane can also be stored.
ii. *Whole Exome Sequencing;*
  The tracking data of WES are the same as the WGS data with additional information for the exome target capture protocol - manufacturer, kit, date performed and by whom.
iii. *RNA-Seq;*
  Sequencing of the transcriptome provides information on gene expression, alternative splicing and gene fusion transcripts. The tracking data are similar to Whole Genome Sequencing with differences in alignment – proportion of reads aligned to introns, exons and ribosomal RNA.
iv. *SNP array;*
  SNP arrays are conducted for single nucleotide polymorphisms detection among individuals. Tracking data include array information, array centre, platform and array quality control as well as results location and analysis algorithms used.
v. *mRNA microarrays;*
  Tracking data of mRNA microarrays for mRNA analysis is largely similar to the SNP array data.
vi. *Array-comparative genomic hybridization (aCGH).*
  aCGH is a method for detection of copy number alterations in the genome. The data include Genomic Hybridisation, dates, aCGH centre and platform, as well as location of results and algorithms used for analysis.
  A *comments* field is available for all the system entities (samples, projects, sources and experiments) to allow any kind of information that is not specified by the database to be entered.

### Sample tracking database schema

#### Sample table

**Figure 1:**
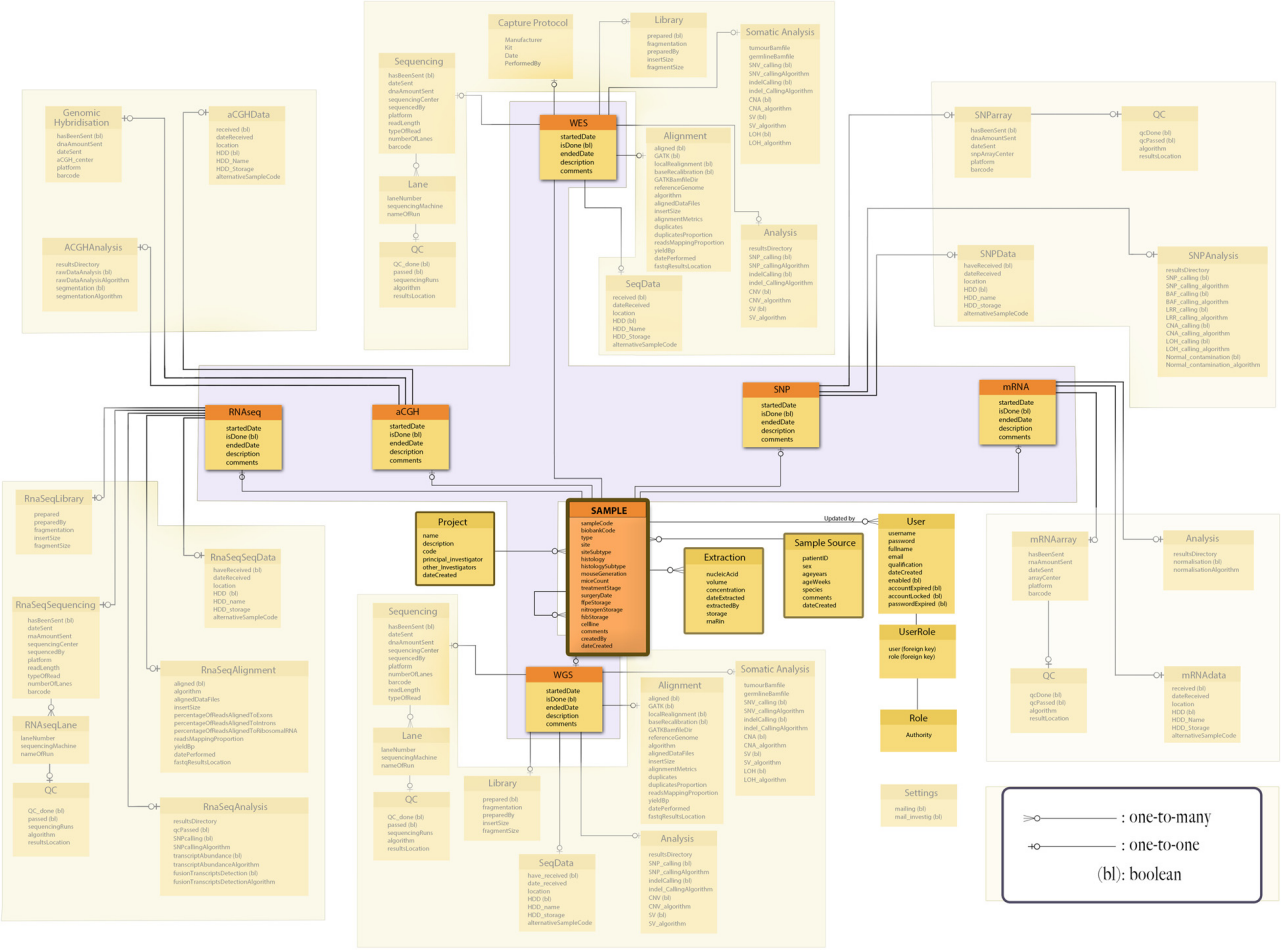
**Summary of the database schema with the** ***sample*** **,** ***patient*** **,** ***project*** **and the six experiments tables (** ***WGS, WES, RNASeq, aCGH, SNPx mRNA*** **) and their connections.** The *User*, *Role* tables and their connection through the *UserRole* table are also shown. The *Sample* table is connected with one-to-many relationship to the *User*, *Patient* and *Project* tables.

Onco-STS is a sample driven application. The *Sample* table (Figure 1) is connected with a one-to-one (1:1) relationship to six tables that correspond to the six different oncogenomic experiments: *WGS*, *WES*, *SNP*, *RNA-Seq, mRNA* and *aCGH*. A one-to-one relationship denotes that each sample record is linked with foreign keys to one particular record from each of the experiment tables.

Each sample belongs to a particular source and project. Consequently, the *Sample* table is also connected with a *Source* and *Project* table. The connection to both tables is through a one-to-many (1:m) relationship, with “many” on the *Sample* side, since each project and source may have multiple different samples (Figure 1).

The users who update the *Sample* table and any other table that is directly or indirectly linked to the *Sample* table are tracked. To accomplish this the *Sample* table is linked with a one-to-many relationship to the *User* table, with “many” on the user’s side, denoting that a sample can be updated by many users (Figure 1). Information for multiple DNA and RNA extractions for each sample is included in the *Extraction* table linked to the *Sample* table (Figure 1).

A sample can be linked to an existing primary sample due to the occurrence of metastases or it can be derived from a primary sample for experiments involving cell-lines, allografts and xenografts. To accomplish this inter-sample relationship the *Sample* table has a self-referencing mechanism, which is used to link samples with each other in a parent–child manner.

#### Experiments

All six experiment tables directly linked to the *Sample* table have exactly the same columns including dates, description and comments. Each of the experiment tables is linked to other tables that hold all the information needed for each experimental record.

i. The *WGS* table has a 1:1 link to a *Sequencing*, *Sequencing Data*, *Library*, *Alignment*, *Analysis* and *Somatic Analysis* table (Additional file 1: Figure S5). The sequencing procedure can be conducted using multiple lanes so the *Sequencing* table has a 1:m link to a *Lane* table. The *Lane* table is 1:1 linked with a *Quality Control* (*QC*) table to store the quality control information of each lane sequenced.
ii. The *WES* table is similar to WGS table with *Sequencing, Sequencing Data, Library, Alignment, Analysis, Somatic Analysis* tables, plus an additional *Capture Protocol* table. The *Lane* and *QC* tables are also the same as in *WGS* (Additional file 1: Figure S6).
iii. The *RNA-Seq* section is similar to *WGS* with some differences in the *Alignment* and *Analysis* tables (Additional file 1: Figure S7).
iv. The *SNP* array table has 1:1 links to an *Array, Data*, and *Analysis* table. The *Array* table is connected with 1:1 relation to a *Quality Control* table to store the quality control information for each SNP array record (Additional file 1: Figure S8).
v. The *mRNA* table has 1:1 links to an *Array, Data*, and *Analysis* table. The *Array* table is connected with 1:1 relation to a *Quality Control* table to store the quality control information for each mRNA array record (Additional file 1: Figure S9).
vi. The *aCGH* table has 1:1 links to an *Array, Data*, and *Genome Hybridization* table (Additional file 1: Figure S10).

The *User* and *Role* tables have records of all the users of the system and the different kind of roles/authorities granted to them. An entire representation of the database schema is shown in Additional file 1: Figure S11.

## Discussion

The sample tracking system developed in this project is a LIMS for oncogenomics studies and facilitates the fast, secure storage and retrieval of sample-associated information. Importantly, details of quality control procedures and analyses of the oncogenomic data are recorded in the system in addition to information on the samples. The web-based service provides advantages over locally accessed software tools as it is platform independent and can be accessed even through mobile devices with a compatible web browser in which case the user interface will be adjusted to the screen size. Additionally, minimal system requirements (and no installation) are needed from the client site and service updates/bug fixes become immediately available to all users. As it is a web application that runs on a server it allows users to access centralized data repositories, which can make collaborations among distant research groups more effective. Furthermore, any new data submitted to the system become instantly available to all scientific staff with access to the system.

We have designed the system to facilitate its use by multiple different users. For example, it is envisaged that new samples could be created by a researcher responsible for a project. Subsequently, information about the sequencing process would be added by a technician or core facility personnel. Thereafter, alignment and analysis tracking would be completed by a bioinformatician. Hence, the system provides a model enabling multiple users to enter data on specific samples. Each one of the experiment steps can be marked as completed which will allow users to check what has been conducted for the experiment and what follows next. Additionally, the progress of each experiment is shown with a progress bar on the sample page to track the progress as it is processed (Additional file 1: Figure S4). The system’s security prevents any unauthorized users from accessing or editing the database.

The web application overcomes many of the drawbacks of having the sample information stored in other forms such as in text file records and allows quick submission, editing and querying of the database.

Onco-STS is limited to allow submission of types of data that have been defined in the system schema. *Comments* fields in all the system sections provide a facility for the addition of further information. Importantly, the system allows linking of samples from the same source (e.g. primary and metastatic tumours from an individual, or cell lines and xenografts derived from a tumour). We have designed the system based on the oncogenomic studies that we conduct routinely in our laboratory. Thus the system is not designed to be a comprehensive resource and is the first version we have constructed. Further versions will include experiment tables for proteomic and epigenomic studies, however these additions are beyond the scope of the present study.

Although Laboratory Information Management Systems (LIMS) already exist, many of them are commercial and not affordable by small research facilities. Additionally, not all of them provide remote and simultaneous access by multiple users. Whilst large scale projects such as the ICGC and TCGA will have dedicated sample tracking solutions, and other research institutes and large laboratories are likely to have implemented systems, many of these are likely to be proprietary or commercial LIMS systems such as the ones offered by Genologics (http://genologics.com/). Although open-source systems for next generation sequencing studies have been published [14-16], our system is tailored for oncogenomic experiments from multiple samples from the same or different sources.

## Conclusions

The current work presents a sample tracking system for oncogenomic studies (Onco-STS) to store these data and make them easily accessible to the researchers who work with the samples. Onco-STS is an easily implemented system based on open-source software and is amenable to future development and modification. Hence it is a good solution for smaller laboratories.

## Availability and requirements

**Project name:** Onco-STS**.**

**Project homepage**: http://sourceforge.net/projects/oncosts/?source=directory.

**Operating system:** Platform independent.

**RAM:** 4 GB.

**Programming language:** Groovy/Grails 2.1.0, Java 1.6.

**Other requirements:** Grails 2.1.0 installation, Java Development Kit (JDK) version 1.6 or above, a Java servlet container like Apache Tomcat, MySQL 5.

**Recommended developing tools:** Groovy/Grails Tool Suite IDE.

**Service usage requirements:** Any operating system with any modern web browser.

**License:** GNU GPL3.

**Any restrictions to use by non-academics:** free to use.

## Additional file

Additional file 1: Figure S1. The homepage of Onco-STS. Purple arrows denote some functional areas of the page. **Figure S2.** A project record view with its attributes and all the samples included in it along with their basic properties. **Figure S3.** Form for new sample submission. **Figure S4.** A sample record view with its properties, links to source and projects it belongs to, and links the six next generation experiments and their sub-sections. The ongoing step for each experiment is show on the experiment progress bar. **Figure S5.** Whole Genome Sequencing table connected with the Sequencing, Sequencing Data, Library, Alignment, Analysis, Lane and Quality Control tables. **Figure S6.** Whole Exome Sequencing table connected with the Sequencing, Sequencing Data, Library, Alignment, Analysis, Capture Protocol, Lane and Quality Control tables. **Figure S7.** RNA Sequencing table connected with the Sequencing, Sequencing Data, Library, Alignment, Analysis, Lane and Quality Control tables. **Figure S8.** SNP array table connected with the Array, Data, Analysis and Quality Control tables. **Figure S9.** mRNA microarrays Array table connected with the Array, Data, Analysis and Quality Control tables. **Figure S10.** Array-Comparative Genomic Hybridization Array table connected with the Genomic Hybridization, Data and Analysis tables. **Figure S11.** The entire database schema of the Onco-STS.

## Abbreviations

TCGA: The Cancer Genome Atlas
ICGC: International Cancer Genome Consortium
SNP: Single Nucleotide Polimorphism
SNV: Single Nucleotide Variations
WGS: Whole Genome Sequencing
WES: Whole Exome Sequencing
SNP: Single Nucleotide Polymorphism Array
aCGH: Array-comparative genomic hybridization
QC: Quality Control
GORM: Grails Object Relational Mapping
LIMS: Laboratory Information Management System
