# Supplementary material for: Onco-STS: a web-based laboratory information management system for sample and analysis tracking in oncogenomic experiments
Source: Source Code Biol Med. 2014 Dec 5;9:25. doi: 10.1186/s13029-014-0025-z (PMC4288629; doi:10.1186/s13029-014-0025-z)
Supplement: Additional file 1: Figure S1. — The homepage of Onco-STS. Purple arrows denote some functional areas of the page. Figure S2. A project record view with its attributes and all the samples included in it along with their basic properties. Figure S3. Form for new sample submission. Figure S4. A sample record view with its properties, links to source and projects it belongs to, and links the six next generation experiments and their sub-sections. The ongoing step for each experiment is show on the experiment progress bar. Figure S5. Whole Genome Sequencing table connected with the Sequencing, Sequencing Data, Library, Alignment, Analysis, Lane and Quality Control tables. Figure S6. Whole Exome Sequencing table connected with the Sequencing, Sequencing Data, Library, Alignment, Analysis, Capture Protocol, Lane and Quality Control tables. Figure S7. RNA Sequencing table connected with the Sequencing, Sequencing Data, Library, Alignment, Analysis, Lane and Quality Control tables. Figure S8. SNP array table connected with the Array, Data, Analysis and Quality Control tables. Figure S9. mRNA microarrays Array table connected with the Array, Data, Analysis and Quality Control tables. Figure S10. Array-Comparative Genomic Hybridization Array table connected with the Genomic Hybridization, Data and Analysis tables. Figure S11. The entire database schema of the Onco-STS. [file 13029_2014_25_MOESM1_ESM.pdf]

Supplementary Figures

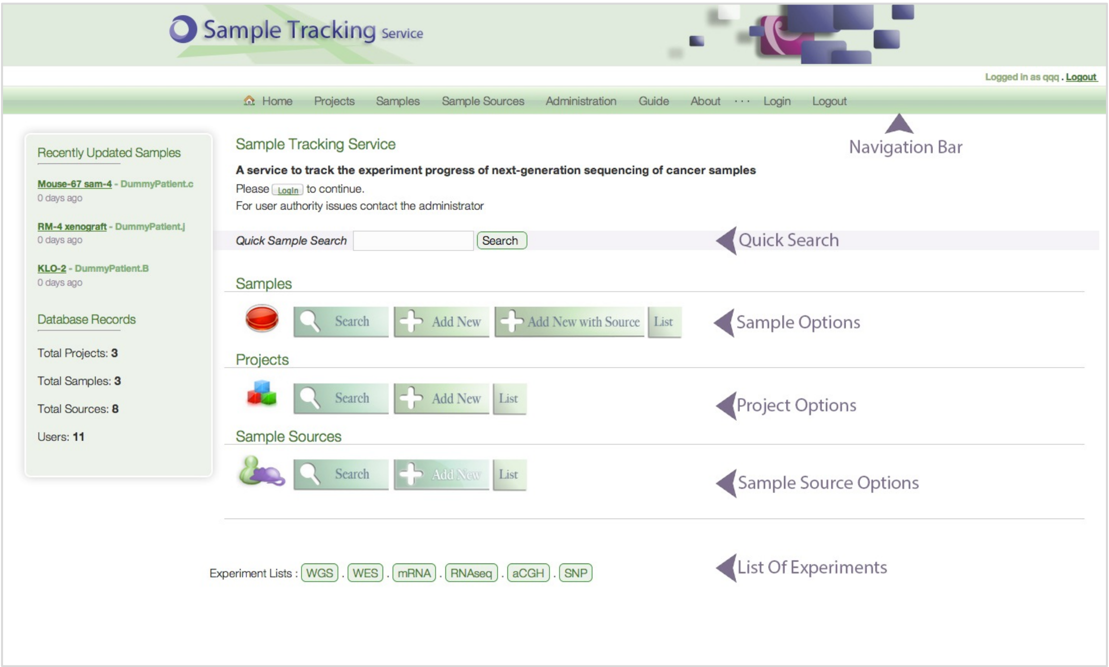

Supplementary Figure 1: The homepage of Onco-STS. Purple arrows denote some functional areas of the page.

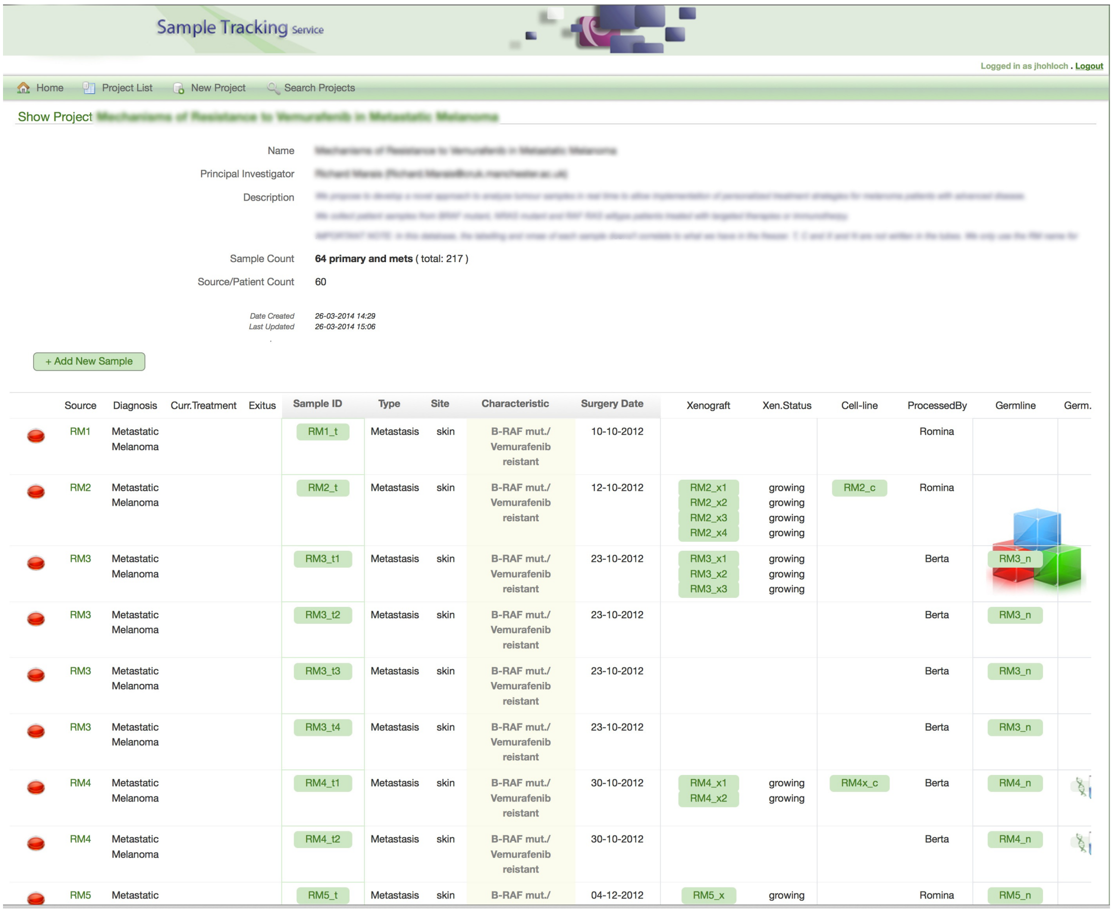

Supplementary Figure 2: A project record view with its attributes and all the samples included in it along with their basic properties.

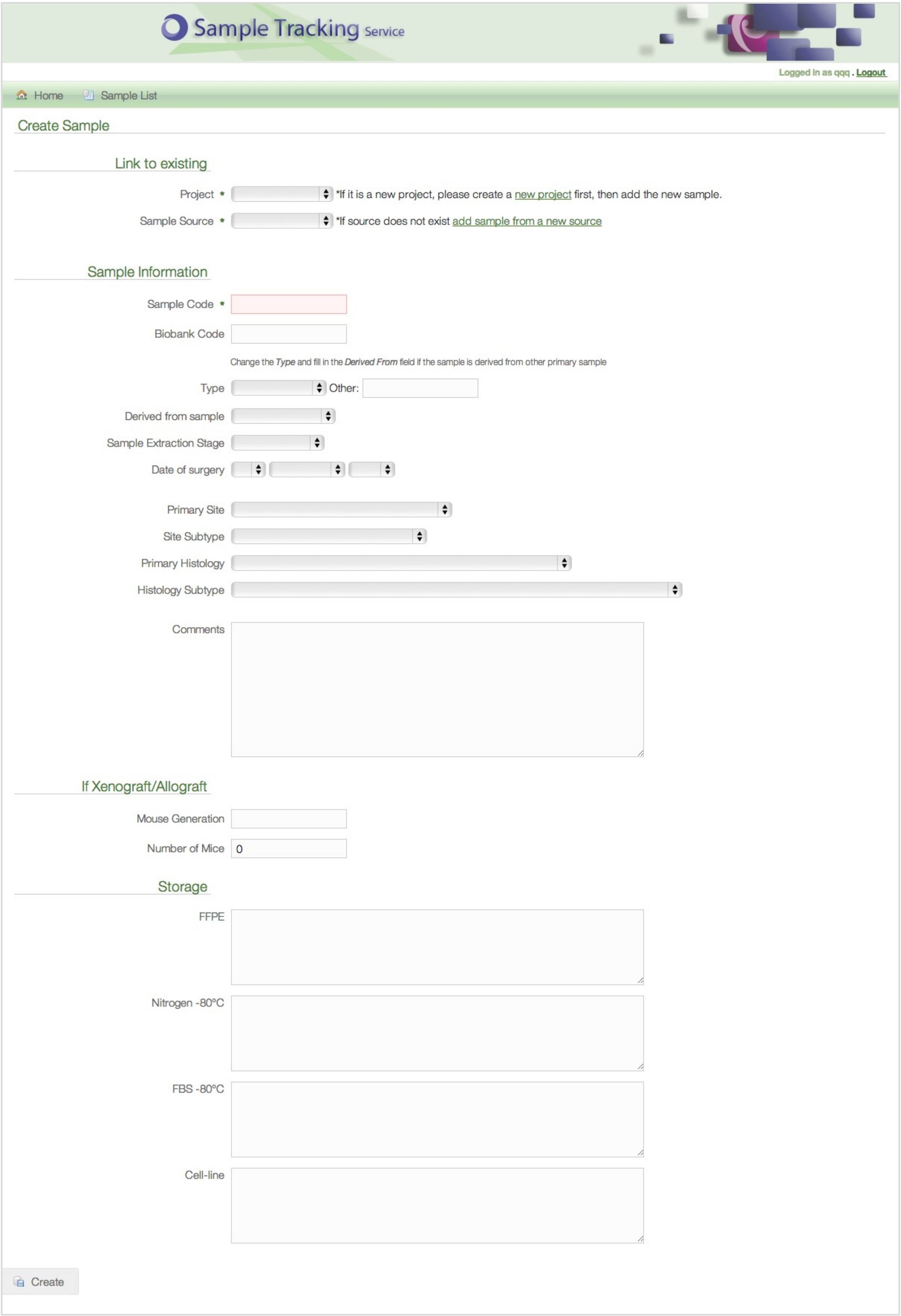

Supplementary Figure 3: Form for new sample submission.

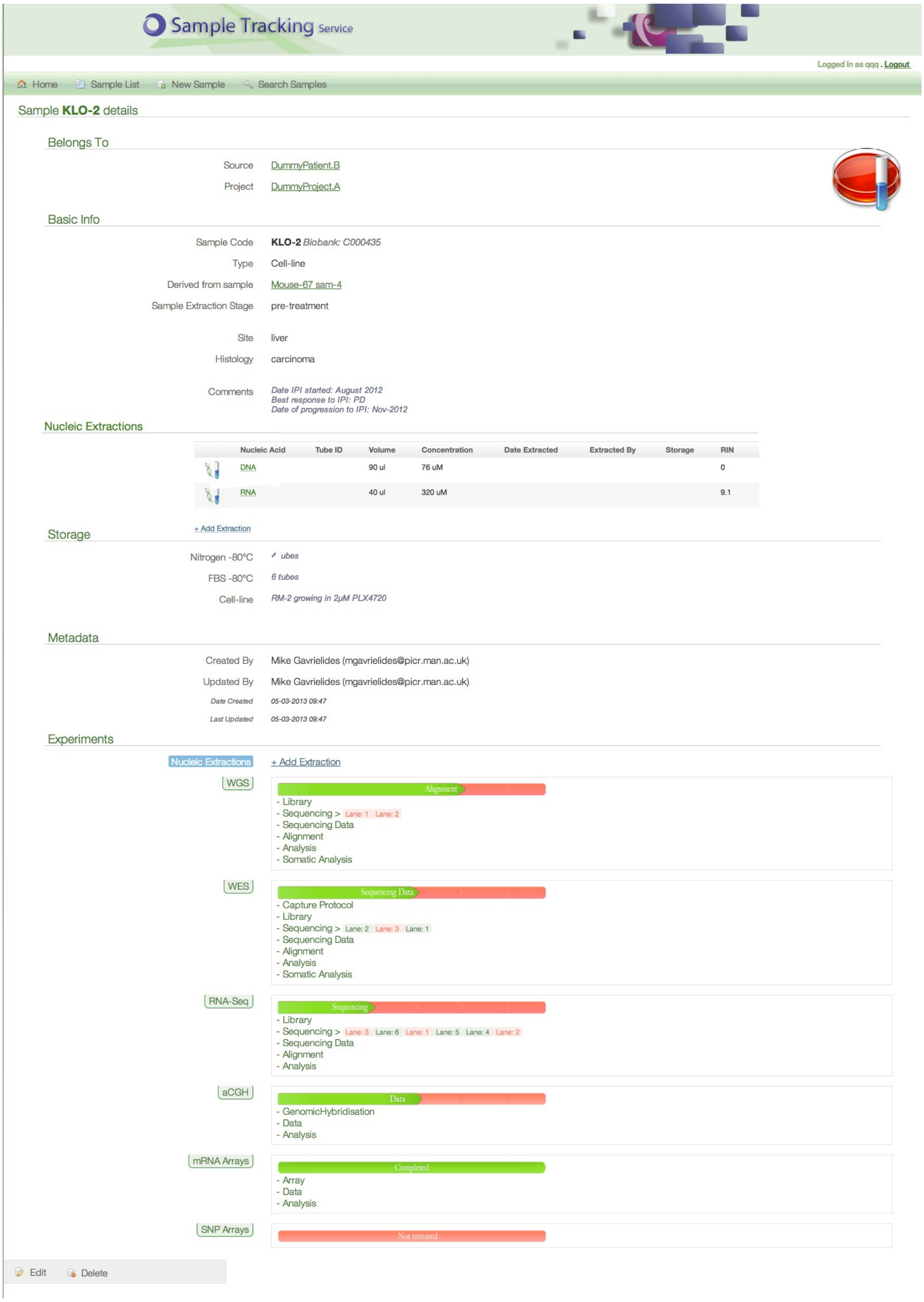

Supplementary Figure 4: A sample record view with its properties, links to source and projects it belongs to, and links the six next generation experiments and their sub-sections. The ongoing step for each experiment is show on the experiment progress bar.

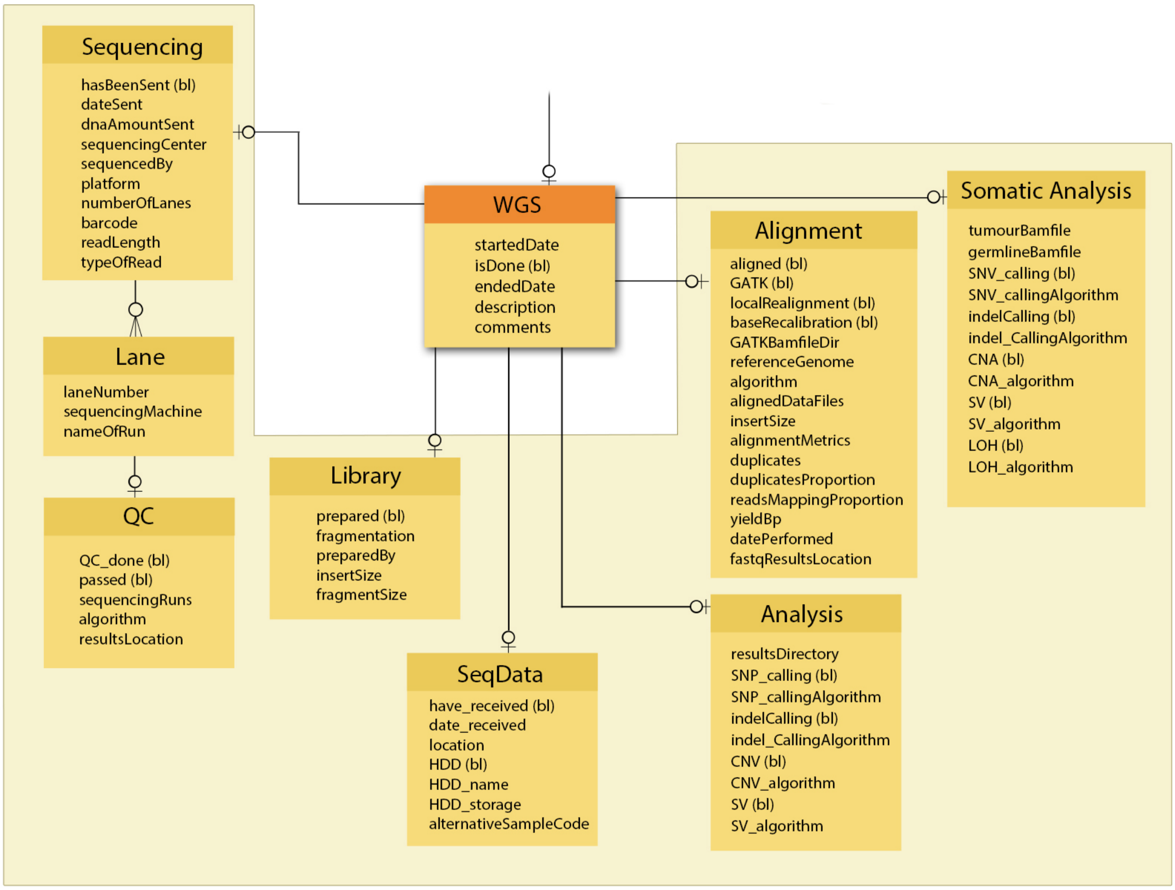

Supplementary Figure 5: Whole Genome Sequencing table connected with the Sequencing, Sequencing Data, Library, Alignment, Analysis, Lane and Quality Control tables.

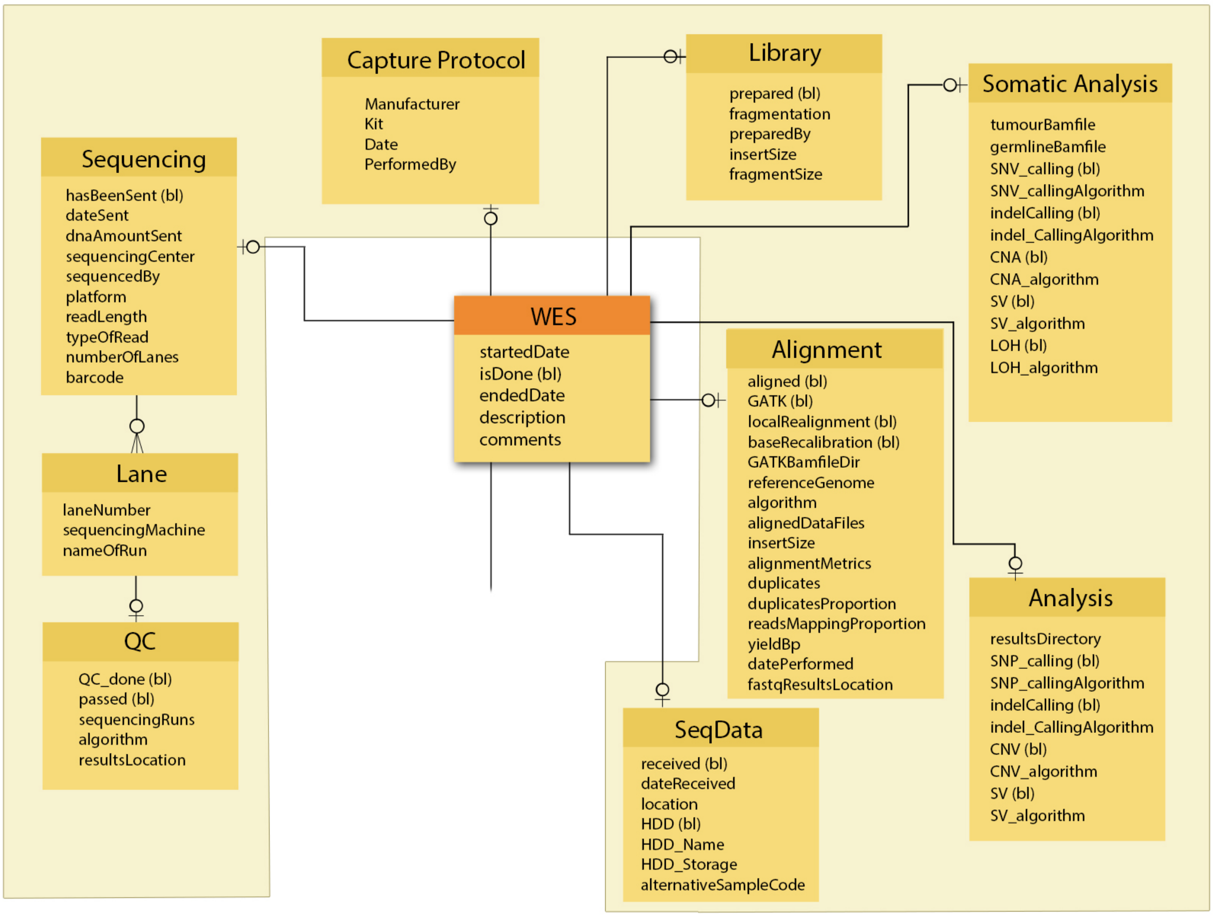

Supplementary Figure 6: Whole Exome Sequencing table connected with the Sequencing, Sequencing Data, Library, Alignment , Analysis, Capture Protocol, Lane and Quality Control tables.

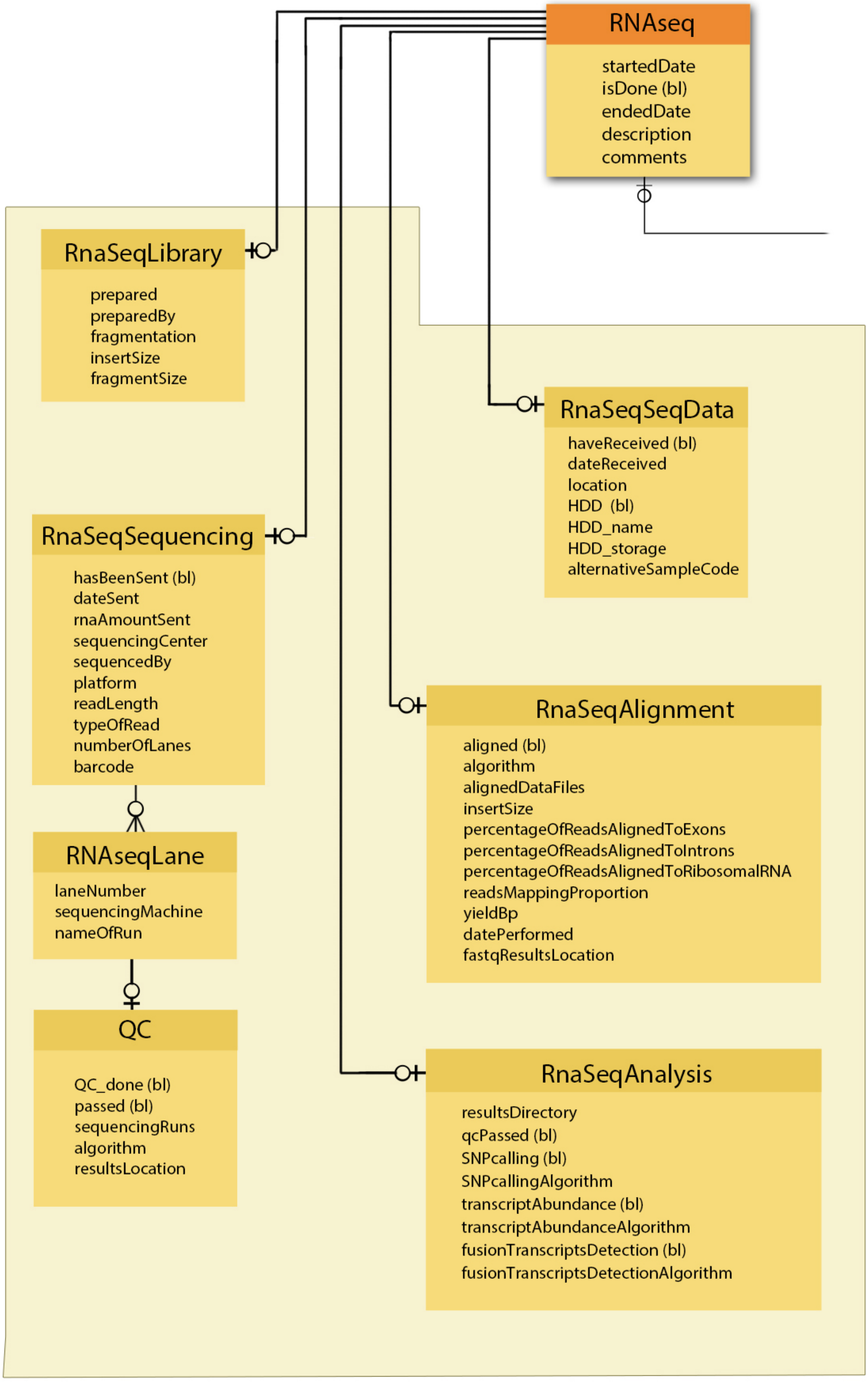

Supplementary Figure 7: RNA Sequencing table connected with the Sequencing, Sequencing Data, Library, Alignment, Analysis, Lane and Quality Control tables.

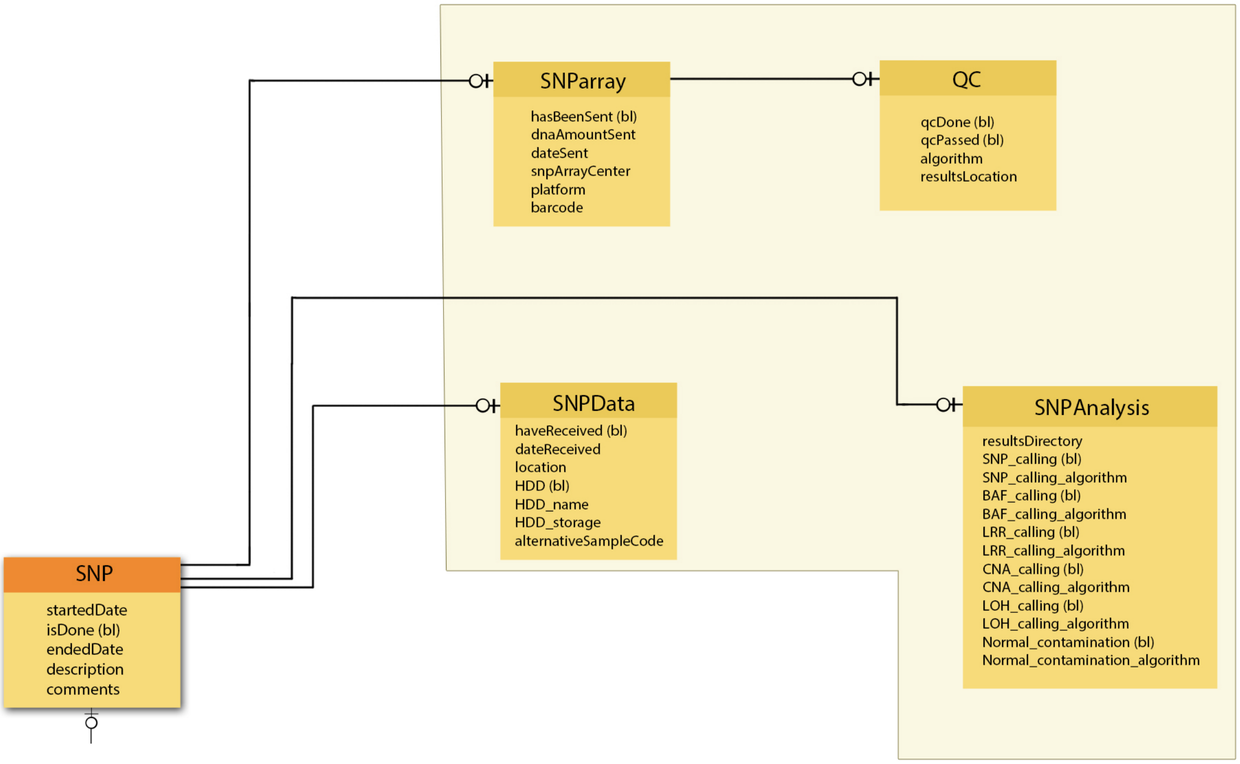

Supplementary Figure 8: SNP array table connected with the Array, Data, Analysis and Quality Control tables.

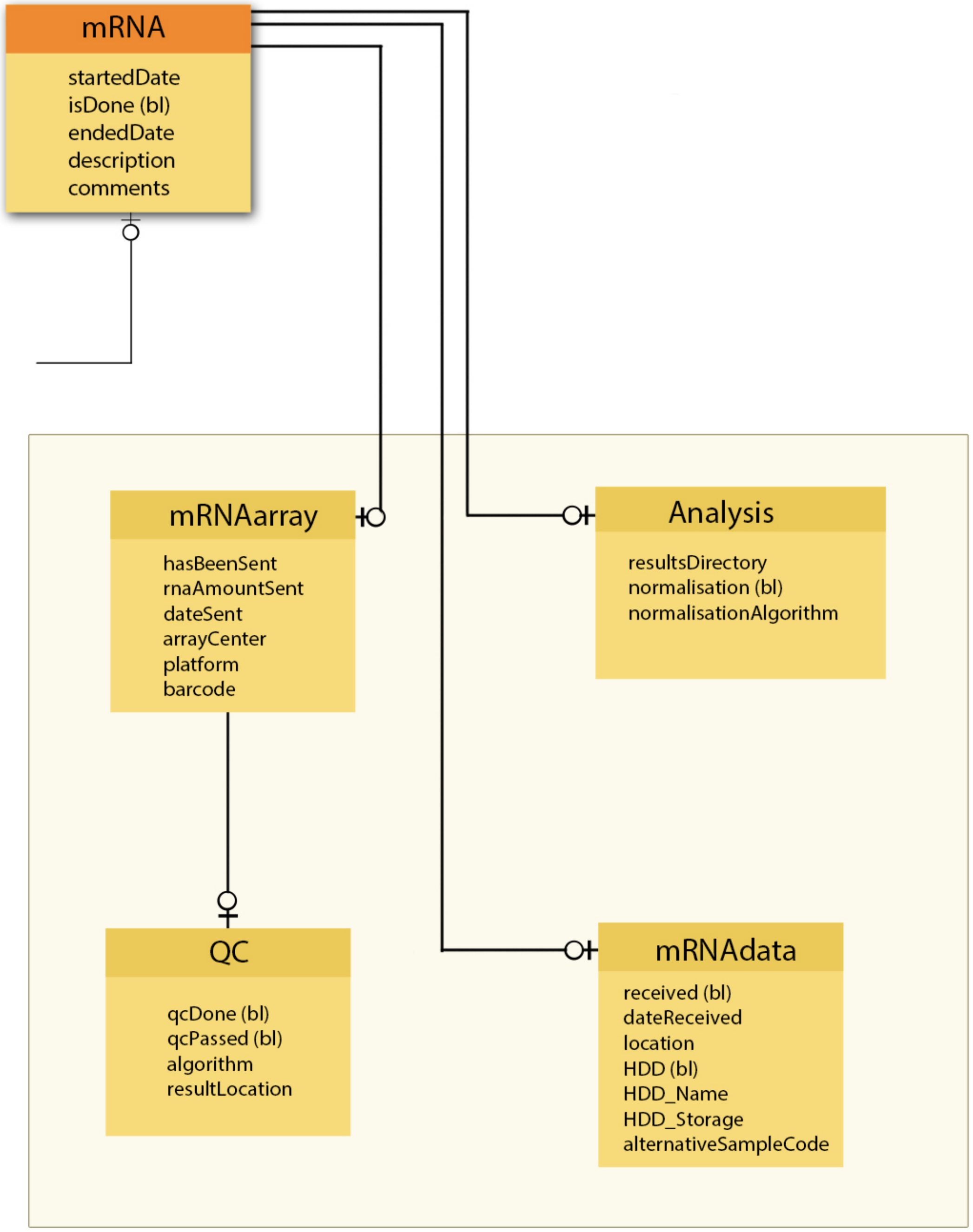

Supplementary Figure 9: mRNA microarrays Array table connected with the Array, Data, Analysis and Quality Control tables.

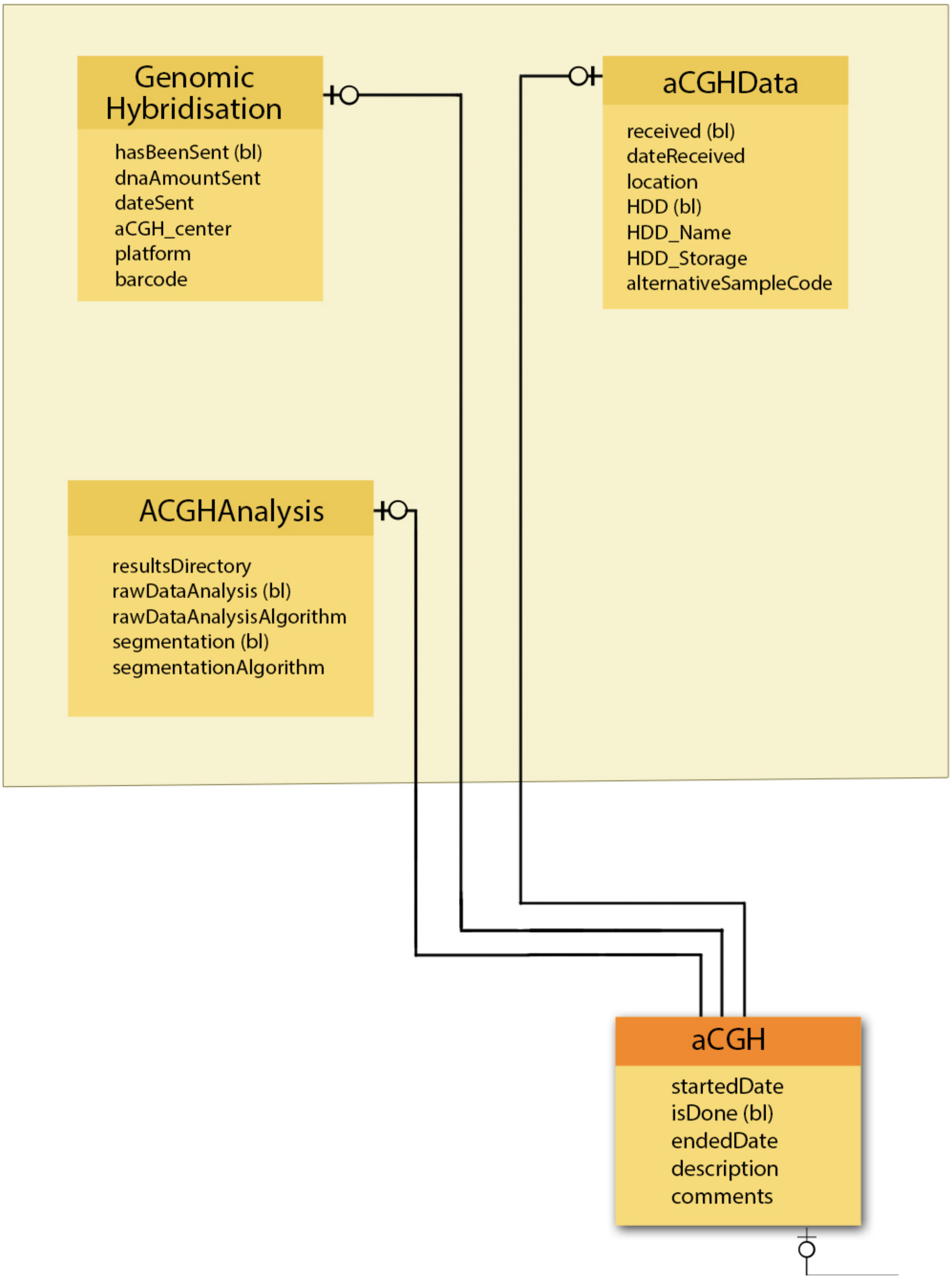

Supplementary Figure 10: Array-Comparative Genomic Hybridization Array table connected with the Genomic Hybridization, Data and Analysis tables.

Sample Tracking Database Schema

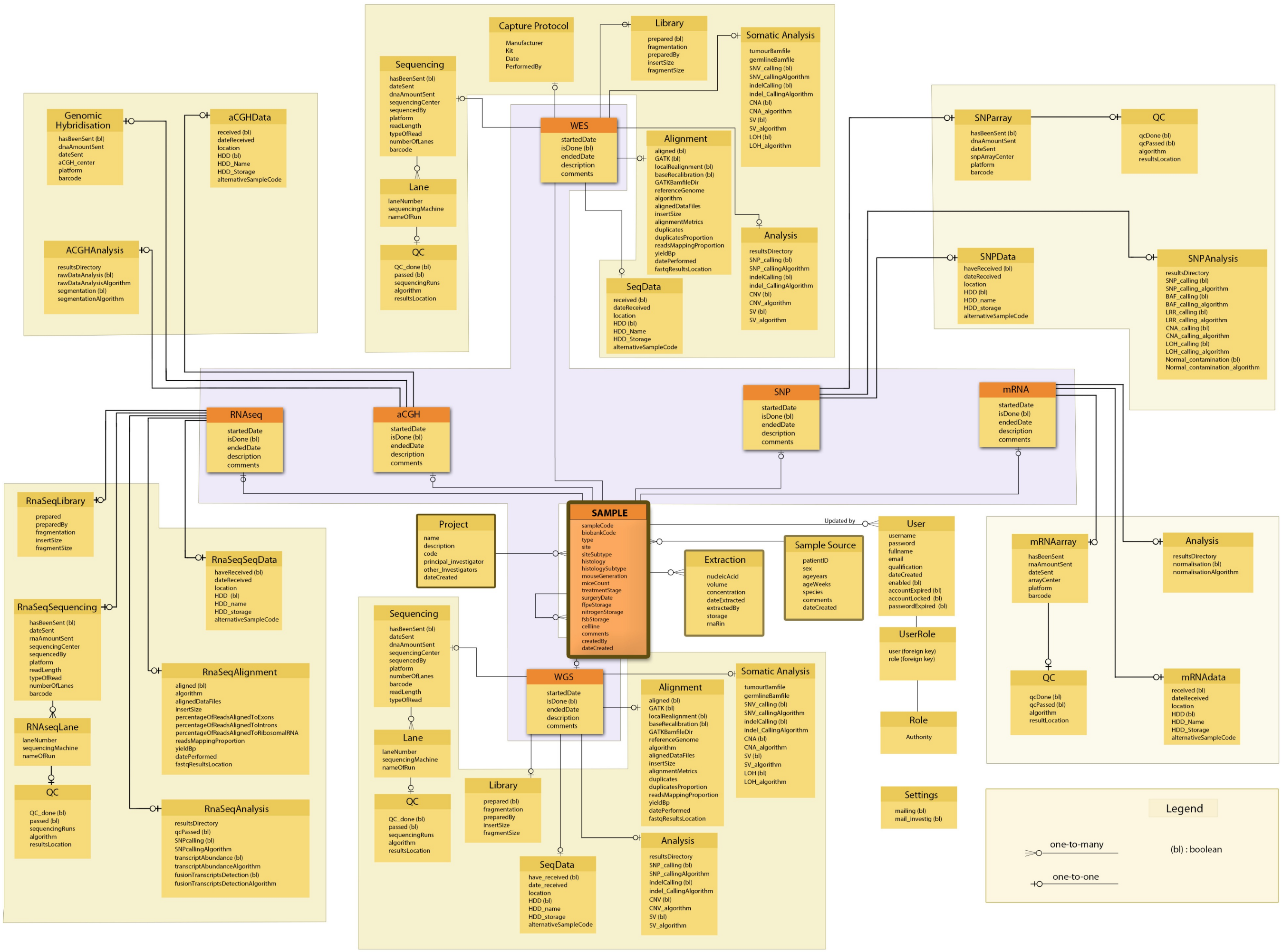

Supplementary Figure 11: The entire database schema of the Onco-STS.
